# The Influence of Severe Plastic Deformation and Subsequent Annealing on the Microstructure and Hardness of a Cu–Cr–Zr Alloy

**DOI:** 10.3390/ma13102241

**Published:** 2020-05-13

**Authors:** Garima Kapoor, Tibor Kvackaj, Anita Heczel, Jana Bidulská, Róbert Kočiško, Zsolt Fogarassy, Dusan Simcak, Jenő Gubicza

**Affiliations:** 1 Department of Materials Physics, Eötvös Loránd University, P.O.B.32, H-1518 Budapest, Hungary; garima_kpr_91@yahoo.com (G.K.); heczel.anita@gmail.com (A.H.); 2 Department of Plastic Deformation and Simulation Processes, Faculty of Materials, Metallurgy and Recycling, Technical University of Košice, 042 00 Košice, Slovakia; tibor.kvackaj@tuke.sk (T.K.); jana.bidulska@tuke.sk (J.B.); robert.kocisko@tuke.sk (R.K.); dusan.simcak@tuke.sk (D.S.); 3 Institute for Technical Physics and Materials Science, Centre for Energy Research, Konkoly-Thege út 29-33, H-1121 Budapest, Hungary; fogarassy.zsolt@energia.mta.hu

**Keywords:** Cu alloy, severe plastic deformation, annealing, microstructure, mechanical behavior

## Abstract

A Cu–1.1%Cr–0.04%Zr (wt.%) alloy was processed by severe plastic deformation (SPD) using the equal channel angular pressing (ECAP) technique at room temperature (RT). It was found that when the number of passes increased from one to four, the dislocation density significantly increased by 35% while the crystallite size decreased by 32%. Subsequent rolling at RT did not influence considerably the crystallite size and dislocation density. At the same time, cryorolling at liquid nitrogen temperature yielded a much higher dislocation density. All the samples contained Cr particles with an average size of 1 µm. Both the size and fraction of the Cr particles did not change during the increase in ECAP passes and the application of rolling after ECAP. The hardness of the severely deformed Cu alloy samples can be well correlated to the dislocation density using the Taylor equation. Heat treatment at 430 °C for 30 min in air caused a significant reduction in the dislocation density for all the deformed samples, while the hardness considerably increased. This apparent contradiction can be explained by the solute oxygen hardening, but the annihilation of mobile dislocations during annealing may also contribute to hardening.

## 1. Introduction

The severe plastic deformation (SPD) process is considered as one of the most effective methods for the production of bulk ultrafine-grained (UFG) and nanostructured materials with novel characteristics [1,2]. Several methods, such as high-pressure torsion (HPT) [3], twist extrusion (TE) [4], multi-directional forging (MDF) [5], equal-channel angular pressing (ECAP) [6] and equal-channel angular rolling (ECAR) [7], cause severe deformation in the structure of metals and alloys by inducing a very large plastic strain. Among these methods, ECAP is known for its simplicity of die design and the high potential for producing relatively large samples. In ECAP, the shear strain is accumulated in the billet during repetitive pressing, which enhances the microstrain with a simultaneous reduction in the size of the grains to a nanocrystalline range [8]. SPD processing was successfully applied to different alloys, such as Cu–Cr–Zr alloys [9,10].

Since single-step processing is not always sufficient to impose huge strains on the material, multi-step processing techniques are usually more effective. According to the previous reports, when materials are pre-processed by non-SPD methods such as rolling, followed by subsequent SPD processing, more pronounced grain refinement could be observed [11,12,13,14]. Conventional rolling at the very low temperature of liquid nitrogen causes the suppression of dynamic recovery in the dislocation structures [15], which could behave as potential recrystallization sites, and the occurrence of grain growth during deformation. Thus, cryorolling is considered as an efficient non-SPD method for achieving a small grain size [6].

The grain size is one of the most important microstructural parameters which governs the physical and mechanical properties of the polycrystalline metals and alloys [16]. The grain refinement causes significant improvement in mechanical properties such as hardness, toughness and strength [17]. Additionally, the type and density of dislocations can also have a significant impact on the properties of nanomaterials. Therefore, it is necessary to explore the relationship between microstructural parameters and their properties. The strength of SPD-deformed materials can be further improved by alloying, as the solute atoms and precipitates have a pinning effect on dislocations and grain boundaries, as shown in previous studies [18,19,20,21,22].

Cu and its alloys are widely known for their excellent applications in several industries, including electrical power, electronics, petrochemicals, transportation, energy, machinery, metallurgy, light, etc. The refinement of the structure to an UFG and nanocrystalline regime further enhances the potential applicability of these alloys. For instance, the deformed Cu-Cr-based alloys exhibit improved strength, wear resistance and fatigue strength; high electrical and thermal conductivity; good thermal stability at high temperatures; and resistance to corrosion [2,23,24,25,26]. The Cu-Cr-based alloys are valuable non-ferrous materials with several applications, such as in integrated circuit lead frames [27,28], high-current connectors [29,30,31], railway contact wires, electrodes for spot welding [4] and switching devices [32].

UFG materials may lose their unique properties during annealing due to the grain coarsening and defect density reduction at high temperatures [33]. Hence, the investigation of these microstructural parameters with annealing at high temperatures is necessary to understand the consecutive impact on the properties of UFG and nanostructured materials [34]. Thus, in this paper, all of the Cu alloy specimens processed under different conditions were further annealed at a high temperature and the comparison of their microstructures, defect structures and hardness before and after annealing is discussed.

This research focuses on the impact of combinations of different SPD processing conditions on the microstructure of a Cu–Cr–Zr alloy. Over the years, several studies have been conducted on the deformation of Cu and its alloys, mostly using a single-step processing technique [8]. However, the microstructure and hardness of Cu–Cr–Zr alloys deformed by the combination of ECAP and rolling (at ambient or liquid nitrogen temperature) have not been investigated until now. All the samples in this study were first processed by ECAP with two different passes, and then the selected samples were subjected to deformation by asymmetric rolling at different temperatures. Furthermore, the changes in the microstructure and defect structure due to annealing were investigated using various characterization techniques. Additionally, the hardness values were also determined for all the samples and then correlated to the microstructural parameters.

## 2. Materials and Methods

### 2.1. Processing of Materials

A Cu–Cr–Zr alloy was used in the present study. The Cr and Zr concentrations were ~1.1 wt.% and 0.04 wt.%, respectively, but the alloy also contained other metallic impurities, such as Al and Fe, with a total concentration of ~0.3 wt.%. The material was heat treated at 1020 °C for 30 min and then water quenched to room temperature (RT). Billets with a circular cross section were manufactured for the ECAP. The diameter and the length of the billets were 10 and 100 mm, respectively. The ECAP was performed for 1 and 4 passes at RT and at a rate of 1 mm/s following the route B_C_. The internal channel angle was 90°, while the angle characterizing the outer arc of curvature at the intersection of the two channels was 20°. Thus, one pass of ECAP corresponded to an equivalent strain of about one.

After the ECAP processing, some samples were further deformed by asymmetric rolling at ambient temperature (297 K) or liquid nitrogen temperature (77 K). Before the first pass and between the consecutive passes of cryorolling, the samples were kept in liquid nitrogen for 10 min. The diameters of the upper and lower working rolls were 40 and 97 mm, respectively. The rolling was performed at a velocity of 0.3 m/s without any lubrication. The initial thickness and width of the rolled plates were 5 and 14 mm, respectively. During each pass of rolling, the material thickness was reduced by approximately 0.83 mm. After three passes of rolling, the thickness decreased to about 2.5 mm, which corresponds to a 50% reduction in thickness.

The samples deformed by ECAP and rolling were subjected to annealing at 430 °C for 30 min in air. Table 1 shows the abbreviations of the different SPD-processed and annealed samples.

### 2.2. Characterization of the Microstructure by Scanning Electron Microscopy

The microstructure was studied in the center of the cross section of the ECAP-processed billets and the rolled plates. The investigation of the microstructures of the SPD-processed and annealed Cu alloys was carried out by electron backscatter diffraction (EBSD) using a FEI Quanta 3D scanning electron microscope (SEM). The energy-dispersive X-ray spectroscopy (EDS) elemental maps were obtained using Hitachi TM4000Plus (Tokyo, Japan) SEM. Before the characterization by EBSD, each sample surface was treated first by mechanical polishing using 600, 1200, 2500 and 4000 grit SiC abrasive papers followed by polishing using a colloidal silica suspension (OP–S) with a particle size of 50 nm. After mechanical polishing, the surface was electropolished using a D2 electrolyte at 16 V and 10 °C for 10 s.

The step sizes during EBSD were between 2.5 and 50 nm, depending on the size of the investigated area. The average grain sizes were obtained from the EBSD images by the investigation of the misorientations in the SPD-processed microstructures. The area-weighted average grain sizes were obtained using the Orientation Imaging Microscopy (OIM) software (version 6).

### 2.3. X-ray Line Profile Analysis

The X-ray line profile analysis (XLPA) measurements were conducted in the center of the cross-section of the samples for the determination of the dislocation density by a high-resolution rotating anode diffractometer (manufacturer: Rigaku, Tokyo, Japan) with CuK_α1_ radiation (wavelength: λ = 0.15406 nm). The two-dimensional imaging plates were used for the detection of Debye–Scherrer diffraction rings. The intensity distribution perpendicular to the rings formed line profiles, which were obtained by integrating the two-dimensional intensity distributions along the rings. These profiles were evaluated using the Convolutional Multiple Whole Profile (CMWP) fitting method [35,36]. The CMWP procedure involves the fitting of the experimental diffraction pattern by the sum of a background spline and the convolution of the instrumental pattern and the theoretical line profiles related to the crystallite size, dislocations and planar faults, if present. The functions of the theoretical line profiles were based on a model of the microstructure where the crystallites have a spherical shape along with a log-normal size distribution and the lattice strains are caused by dislocations. The instrumental broadening was negligible for all the investigated samples, compared to the physical peak broadening due to the UFG microstructure of the samples.

The microstructures of all the SPD-processed Cu–Cr–Zr samples were studied by XLPA before and after annealing. The following parameters of the microstructure were obtained after the evaluation: the area-weighted mean crystallite size (<x_area_>), the average dislocation density (*ρ*) and the dislocation arrangement parameter (*M*). The parameter *M* is a dimensionless quantity which describes the arrangement of dislocations. The lower *M* value corresponds to the higher shielded strain field of dislocations; the arrangement of dislocations into low energy configurations, such as low angle grain boundaries or dipoles, is reflected by the reduction in the value of *M*. The area-weighted mean crystallite size was determined as <x_area_> = *m*·exp (2.5 *σ*^2^), where *m* is the median and *σ*^2^ is the log-normal variance in the crystallite size distribution.

### 2.4. Transmission Electron Microscopy Study

The microstructure of a few selected samples was studied by scanning transmission electron microscopy (STEM). These experiments were carried out by an aberration-corrected Titan-Themis microscope (manufacturer: FEI, Hillsboro, OR, USA) at 200 keV. For EDS characterization, a Super-X detector was used. The TEM samples were prepared by focused ion beam (FIB) milling.

### 2.5. Hardness Testing

The microhardness of the Cu alloys processed under different conditions was determined before and after annealing using a Zwick Roell ZHμ (Kennesaw, GA, USA) hardness tester with a Vickers indenter. For these investigations, a fixed load of 500 g was applied for a constant dwell time of 10 s. On average, the measurements of each sample were repeated 10 times at different positions.

## 3. Results

### 3.1. Microstructure Development During ECAP and Subsequent Rolling

**Figure 1:**
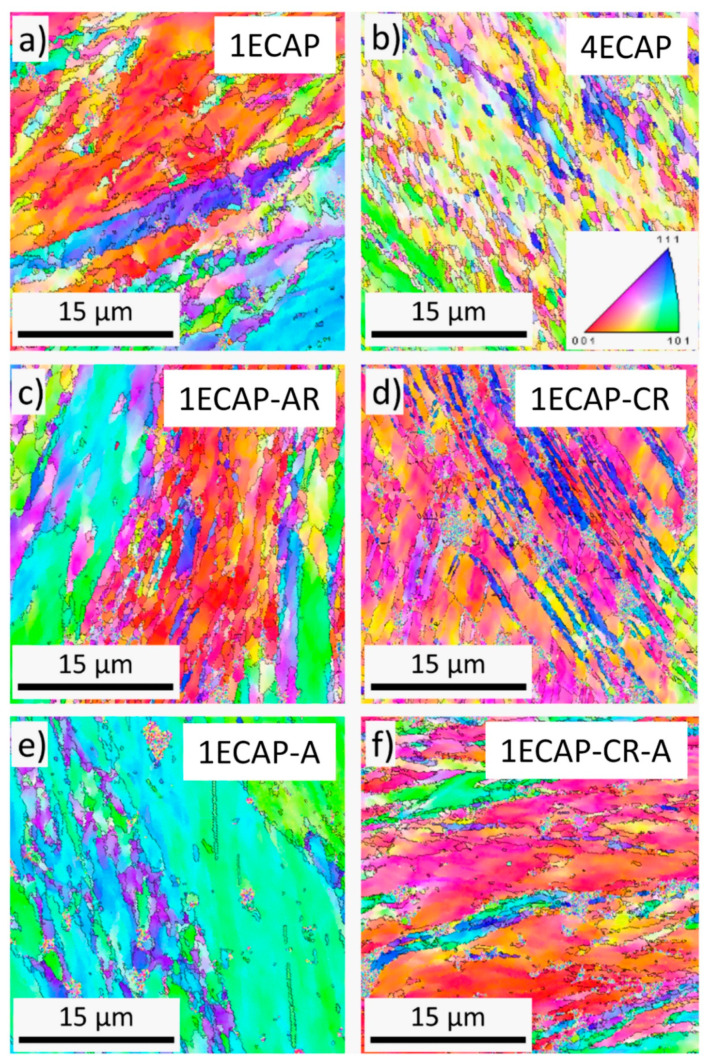
Electron backscatter diffraction (EBSD) grain orientation maps for samples 1ECAP (**a**), 4ECAP (**b**), 1ECAP–AR (**c**), 1ECAP–CR (**d**), 1ECAP–A (**e**) and 1ECAP–CR–A (**f**). The color code for the different orientations is shown in (**b**). The boundaries with misorientations higher than 2° are indicated by black lines.

The grain size of the initial alloy before SPD processing was about 40 µm. The dislocation density in this material was under the detection limit of the XLPA method (<10^13^ m^−2^). The grain structure of the SPD-processed samples was studied by EBSD. Typical EBSD grain orientation maps for some selected specimens are shown in Figure 1.

The area-weighted average grain/subgrain size values were determined with the misorientation angle limit of 2° and are listed in Table 2.

This angle limit value was selected because it has been shown that low-angle boundaries with misorientation angles above 2°–3° strengthen the material, similar to conventional high-angle grain boundaries [37]. Table 2 shows that after one pass of ECAP, the average grain/subgrain size was 10 ± 2 µm, which decreased to 4 ± 1 µm when four passes were completed. Additional rolling at ambient and cryogenic temperatures yielded a two- and four-fold reduction in the grain/subgrain sizes, respectively. Similar grain sizes were observed by EBSD for other ECAP-processed Cu samples in previous publications [38,39,40,41].

**Figure 2:**
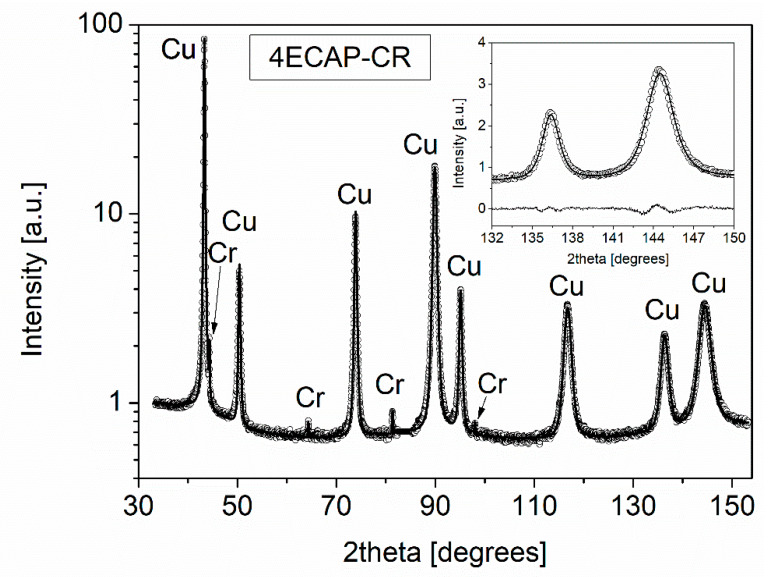
Convolutional Multiple Whole Profile (CMWP) fitting of the XRD pattern taken for the sample 4ECAP–CR. The open circles and solid line represent the measured and fitted diffractograms, respectively. The intensity is in a logarithmic scale. The inset shows a magnified part of the pattern in a linear intensity scale. In this figure, the difference between the measured and fitted patterns is shown at the bottom.

The crystallite size (or diffraction domain size) and dislocation density were determined by XLPA. As an example, Figure 2 shows a CMWP fitting on the diffraction pattern taken for the sample 4ECAP–CR. 

The crystallite size and dislocation density values obtained by the CMWP fitting are listed in Table 2. It can be seen that upon increasing the number of ECAP passes from one to four, the value of dislocation density increased from (17 ± 2) × 10^14^ m^−2^ to (25 ± 2) × 10^14^ m^−2^. The value of dislocation arrangement parameter *M* decreased from 3.3 ± 0.3 to 2.2 ± 0.3 upon increasing the number of passes from one to four, implying a higher shielded strain field of dislocations in the four ECAP-processed sample.

With additional cryorolling at liquid nitrogen temperature, the dislocation density value became more than double for the one ECAP-processed sample. However, when rolling was carried out at RT instead of liquid nitrogen temperature after one ECAP pass, the dislocation density practically remained unchanged. Similar trends were observed for samples deformed with four ECAP passes. The dislocation density value of the four ECAP-processed alloy increased from (25 ± 2) × 10^14^ m^−2^ to (39 ± 5) × 10^14^ m^−2^ after subsequent cryorolling, but did not change by rolling at RT. The increase in dislocation density with the increasing number of ECAP passes from one to four is accompanied by the reduction in crystallite size from 84 ± 9 nm to 57 ± 6 nm. Subsequent rolling at RT did not cause a significant change in the crystallite size, irrespective of the number of ECAP passes. When the ECAP-deformed specimens were processed by cryorolling, the value of the crystallite size considerably decreased for the 1ECAP–CR sample, but almost no change was observed for the 4ECAP–CR sample. It is noted that the crystallite size determined by XLPA is much smaller than the grain/subgrain size obtained by EBSD. This difference is caused by the fact that XLPA is more sensitive to misorientations than EBSD, and XLPA detects two volumes as different crystallites even if the misorientation is only in the order of magnitude of 0.1°.

**Figure 3:**
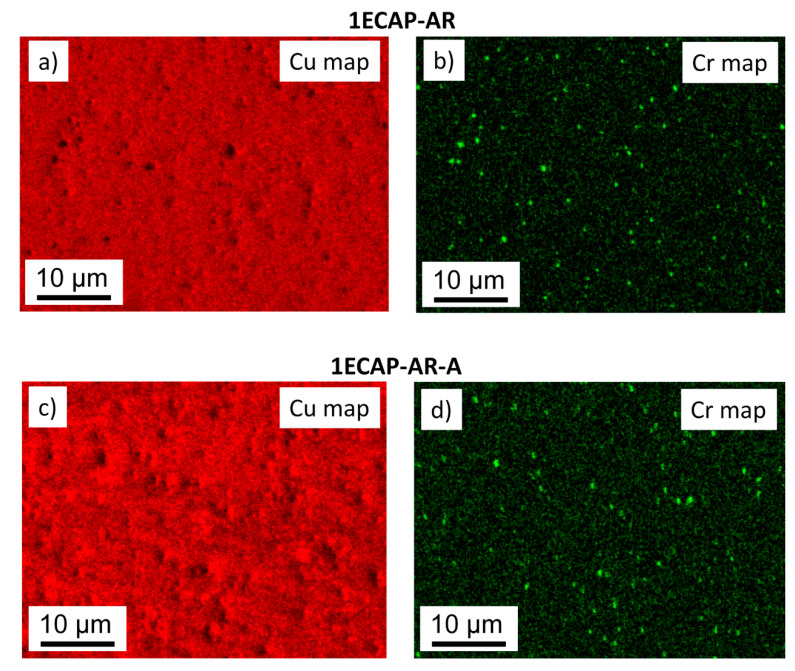
SEM-EDS elemental maps for Cu (**a**,**c**) and Cr (**b**,**d**) in the cases of the samples 1ECAP–AR (**a**,**b**) and 1ECAP–AR–A (**c**,**d**).

As an example, the elemental maps of Cu and Cr obtained by SEM-EDS investigations can be seen in Figure 3 for the 1ECAP–AR and 1ECAP–AR–A samples.

The EDS investigation confirmed the presence of Cr particles in all the samples with an average size of 1 μm. The existence of the Cr phase was further confirmed by XRD (see Figure 2), which revealed that the fraction of the Cr phase in the Cu alloys was about 0.4 ± 0.2% for all SPD-processed and annealed samples.

### 3.2. Changes in the Microstructure During Annealing of the SPD-Processed Samples

After preliminary SPD processing, all the samples were annealed at 430 °C for 30 min in air. The X-ray diffraction (XRD) studies showed a considerable decrease in dislocation density and increased crystallite size with annealing. Both the reduction in dislocation density and increase in crystallite size is more pronounced for samples processed by four ECAP passes compared to one ECAP pass, as shown in Table 2. The increase in the dislocation arrangement parameter was also observed after heat treatment in all the investigated samples.

Annealing of the samples cryorolled after one and four ECAP passes resulted in the lowering of the dislocation density value by ~50% and ~36%, respectively. When the ECAP-deformed samples rolled at RT were annealed to 430 °C, the decrease in the dislocation density for both one and four ECAP passes was less significant compared to the cryorolled specimens. Relatively, a more substantial impact of annealing occurred on the dislocation density in the case of 4ECAP–AR than the 1ECAP-AR sample. Additionally, a considerable difference in crystallite size was also observed before and after annealing for specimens that were subjected to rolling at RT.

**Figure 4:**
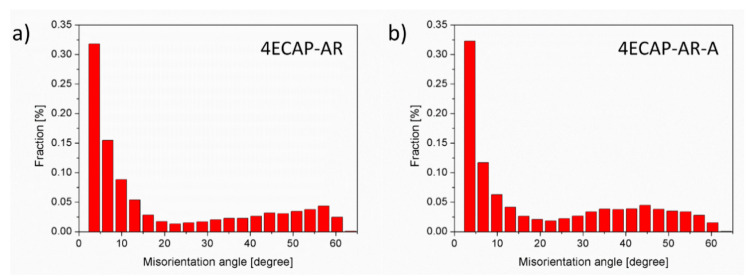
Grain boundary misorientation distributions measured by EBSD for the samples 4ECAP–AR (**a**) and 4ECAP–AR–A (**b**).

The grain boundary misorientation distributions were determined from the EBSD images. A comparison of the misorientation distributions is shown in Figure 4 for the samples 4ECAP–AR and 4ECAP–AR–A.

**Figure 5:**
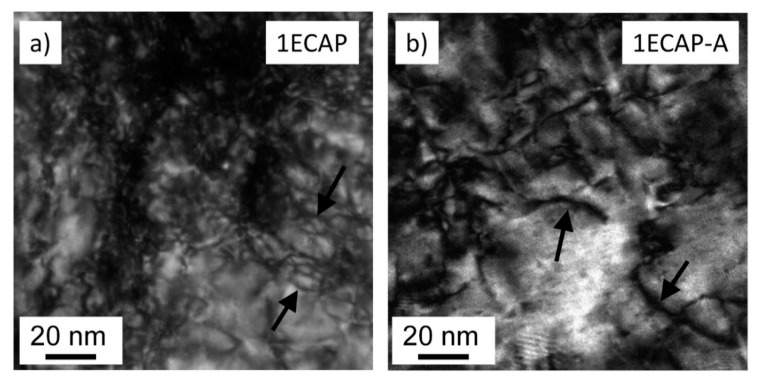
TEM bright-field images taken for the samples 1ECAP (**a**) and 1ECAP–A (**b**). Some dislocations are indicated by black arrows.

It is revealed that annealing did not result in any notable changes in the grain boundary misorientation distribution. XRD investigations showed that no change in the Cr fraction occurred even after the heat treatment. Figure 5 shows the comparison of the bright-field TEM images for the 1ECAP and 1ECAP–A samples which were taken from a <422> type orientation.

The curves with a dark contrast indicate dislocations in the Cu matrix (some of them are marked by black arrows). The decrease in the dislocation density after annealing is evident by comparing the images before and after the heat treatment. It is noted that the TEM images shown in this study are not suitable for the quantitative evaluation of the dislocation density in the whole samples, since this method investigates a very small volume of the material. Therefore, the evolution of the dislocation density during SPD and the subsequent annealing was determined instead by the XLPA method, which studied a volume about seven orders of magnitude larger than the TEM did.

### 3.3. Effect of SPD-Processing and Subsequent Annealing on the Hardness

**Figure 6:**
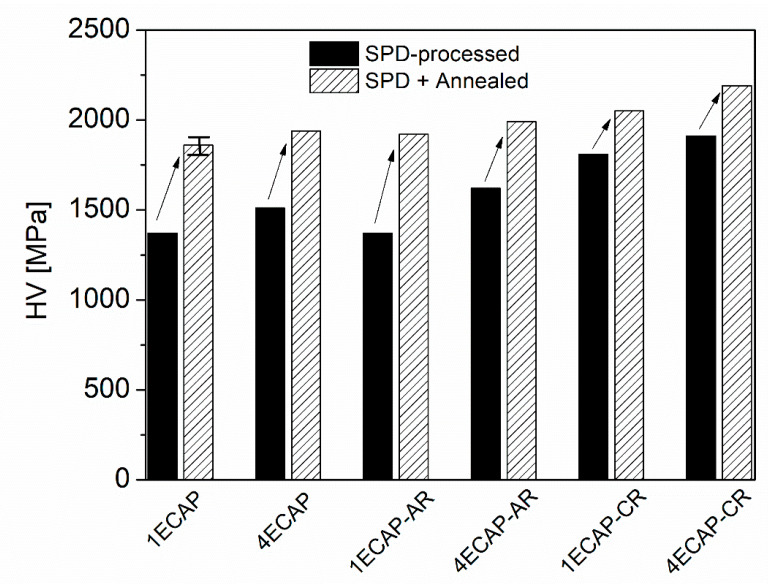
The Vickers hardness (HV) values for the samples processed by equal channel angular pressing (ECAP) and subsequent rolling, as well as for the post-annealed specimens. The arrows indicate the increase in the hardness due to the heat treatment. The error is illustrated by the bar at the column related to the sample annealed after one ECAP.

The hardness of the initial sample before the SPD processing was 490 ± 30 MPa. Figure 6 shows the hardness values for the different SPD-processed samples before and after annealing. To identify the presence of inhomogeneity along the cross-sectional surface of the samples, the hardness values were measured randomly at different positions on the surface of each sample; the calculated average values are given in Figure 6.

The error of hardness was about 2% for all samples, i.e., a considerable difference in the hardness along the surface was not observed for all the studied samples. It can be seen from Figure 6 that the hardness value in the ECAP-deformed specimens increased by ~9% with increasing the number of ECAP passes from one to four. The subsequent rolling at RT further enhanced the hardness of the sample processed by four ECAP but did not cause any change for the one ECAP sample. Similar to the dislocation density, cryorolling after ECAP processing resulted in considerably higher hardness values than deformation by rolling at RT for both of the ECAP passes. Moreover, the annealing led to an increase in hardness compared to the values obtained for the deformed samples before the heat treatment. This increase is less pronounced for the ECAP-processed samples subjected to cryorolling. Nevertheless, among all the samples the maximum value of hardness was obtained for the annealed state of the sample 4ECAP-CR.

## 4. Discussion

Figure 6 unambiguously reveals that annealing at 430 °C for 30 min in air caused a significant hardening for all the SPD-processed samples. This increase in the hardness varied between 13% and 40%, depending on the preliminary SPD processing. As the diffraction domain size obtained by XLPA and the subgrain size determined by EBSD did not change or slightly increased during annealing (see Table 2), the Hall–Petch effect cannot be the reason for the observed hardening. In addition, the heat treatment did not cause a significant change in the grain boundary misorientation distribution, as shown in Figure 4, therefore the hardness increase cannot be explained by the change in the grain boundary character. A considerable change in the fraction of the Cr phase during annealing was not observed by XRD; it remained 0.4 ± 0.2% for all the specimens. The size of the Cr particles was about 1 µm both before and after the heat treatments for all the SPD-processed samples (as an example, a pair of samples is shown in Figure 3). Thus, change in the precipitate structure cannot be the reason for the detected hardening.

**Figure 7:**
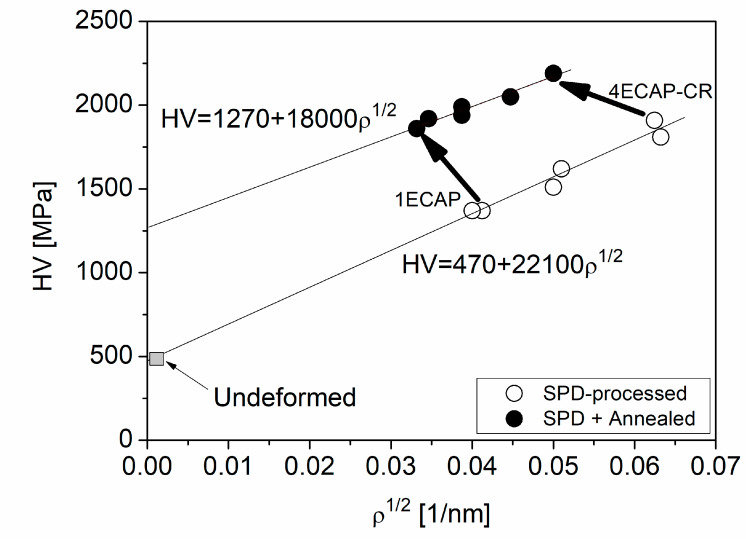
Taylor plotting of the hardness (HV) versus the square root of the dislocation density (*ρ*) for the severely deformed and post-annealed samples as indicated by open and solid circles, respectively. The two thick arrows show the hardening caused by the annealing of the samples subjected to the least (1ECAP) and the most (4ECAP–CR) severe deformation. The Taylor formulas obtained by separate fitting on the open and solid circles are also shown. The grey square corresponds to the initial undeformed material.

Former studies [42] have shown that heat treatments performed at moderate temperatures (at the homologous temperatures 0.35–0.45 × *T*_m_, where *T*_m_ is the melting point) can cause hardening in UFG and nanomaterials. In SPD-processed samples, one possible reason for this anneal hardening is the annihilation of mobile dislocations and/or their clustering, which caused a more difficult initiation of plastic deformation after the heat treatment [42,43,44]. Indeed, in our samples the dislocation density decreased during annealing and, additionally, the dislocation arrangement parameter increased (see Table 2), which suggests significant changes in the dislocation structure. For the analysis of the effect of the dislocations on the hardness, a Taylor plot was constructed in Figure 7 using the following formula:

(1) $$HV=HV_{0}+3\alpha M^{T}Gb\rho^{1/2},$$

where $HV_{0}$ accounts for the hardness contributions coming from other sources than dislocation-dislocation interaction (e.g., solute hardening and precipitate hardening). In Equation (1), α is a parameter describing the strength of the interaction between dislocations and $M^{T}\;$is the Taylor factor, which is 3.06 for untextured polycrystalline fcc materials. This value will be used in this analysis, since a strong texture was not observed in the studied samples. The shear modulus and the magnitude of the Burgers vector in Equation (1) for Cu are *G* = 47 GPa and *b* = 0.256 nm, respectively.

The fitting of Equation (1) on the data related to the SPD-processed samples (open symbols in Figure 7) yielded a $HV_{0}$ = 470 ± 110 MPa and a 3αMGb = 22,100 ± 2200 MPa·nm. It should be noted that the value of $HV_{0}$ obtained by fitting was in good agreement with the hardness measured in the initial sample before SPD processing (490 ± 30 MPa). Using the parameter values given in the previous paragraph, 0.2 was obtained for α, which is in good agreement with the value published formerly for SPD-processed Cu [8]. A similar fitting to the points in Figure 7 obtained for the annealed samples (solid circles) resulted in a $HV_{0}$ = 1270 ± 80 MPa and a 3αMGb = 18,000 ± 1900 MPa nm. It can be seen that the heat treatment at 430 °C for 30 min in air caused a significant increase in $HV_{0}$, while the slope only slightly decreased. Indeed, the value of α was reduced from 0.2 to 0.16 during annealing. This decrease is in accordance with the increase in the dislocation arrangement parameter *M* (see Table 2). Indeed, former studies [45,46] have proved that a higher value of *M* corresponds to a less clustered dislocation arrangement, which usually yields a lower dislocation hardening (i.e., a smaller value of α). The decrease in the dislocation arrangement parameter during annealing can be explained by the annihilation of dislocation dipoles since, in this configuration, the dislocation strain field is strongly shielded by the other dislocation in the dipole. However, this effect on the hardness is less pronounced than the increase in $HV_{0}$. It is noted that the Hall–Petch term was not included in Equation (1), since the grain/subgrain sizes were relatively large (see Table 2).

The parameter $HV_{0}$ in Equation (1) describes the combined hardening effects of lattice friction, solute atoms and precipitates. The amount of Cr precipitates and their size did not change during annealing, therefore the contribution of precipitates to the hardness was similar before and after annealing. Therefore, an increase in the lattice friction or an enhanced solute hardening might have caused the rise in hardness after the heat treatment. The former effect is practically the increase in threshold stress required for the movement of a dislocation in an obstacle-free crystal. This effect may be caused by the annihilation of mobile dislocations, and therefore the initiation of the glide of the rest of the dislocations is more difficult during the hardness test. However, this effect can cause only a maximum 20% increase in hardness [42] while in our case the $HV_{0}$ was enhanced by a factor of about three (see Figure 7). Therefore, the possible increase in the friction stress cannot be the only reason for the hardening observed after annealing. Another possible reason for the enhanced hardness is the increased solute hardening caused by the infiltration of oxygen during the heat treatment performed in air. Indeed, TEM-EDS on the sample 1ECAP indicated that the average oxygen concentration increased to 5 ± 2 at.% during annealing, while in the initial ECAP-processed state the oxygen content was under the detection limit of 0.2 at.%. This large increase in oxygen concentration could be responsible for the three-fold increase in $HV_{0}$. Indeed, a former study on Ti proved that when the oxygen concentration increased from 0.1 to 0.3 wt.%, the flow stress at a plastic strain of about 8% was enhanced from about 480 to 800 MPa [44]. This strain value was selected for the comparison of the flow stresses at different oxygen contents, as hardness testing causes an 8% extra plastic strain in the material. It was revealed that oxygen-induced hardening in Ti was caused by the interaction between dislocations and solute oxygen atoms. Namely, the oxygen interstitials were segregated to the tension side of the edge dislocation core [46]. In the case of screw dislocations, there is a short-range but strong repulsion between dislocations and interstitial oxygen atoms due to the distortion of the interstitial sites at the dislocation core [47]. Therefore, for both edge and screw dislocations, oxygen interstitials hinder the motion of dislocations, thereby resulting in strengthening. This effect can also work in other materials, such as Cu, as stated in [48]. Accordingly, the nearly three-fold enhancement of $HV_{0}$ for the present Cu alloy could be caused by the large increase in oxygen concentration during annealing. For a general confirmation of the oxygen-induced hardening effect, additional oxygen concentration measurements are planned for all the SPD-processed and annealed Cu-Cr-Zr samples (not only for the 1ECAP specimen).

## 5. Conclusions

The evolution of the microstructure and hardness in a Cu–Cr–Zr alloy during SPD processing and subsequent annealing at 430 °C for 30 min was studied. The following conclusions were drawn from the results:

1. ECAP processing for one pass resulted in a dislocation density of ~17 × 10^14^ m^−2^ and a crystallite size of ~84 nm. The dislocation density increased to ~25 × 10^14^ m^−2^, while the crystallite size decreased to ~57 nm when the number of passes was raised to four. Additional rolling at ambient temperature did not yield a further change in the dislocation density and crystallite size. At the same time, cryorolling after ECAP resulted in an increase in the dislocation density to ~40 × 10^14^ m^−2^ and a small crystallite size of about 50 nm, irrespective of the number of ECAP passes.
2. The heat treatment caused a significant reduction in the dislocation density and a change in the dislocation configuration, leading to a less shielded strain field of dislocations. This could be a result of the annihilation of dislocation dipoles during annealing. In addition, the oxygen content of the samples considerably increased, since the heat treatment was carried out in air.
3. The hardness evolution during the SPD processing and subsequent annealing obeyed the Taylor relationship, indicating that the interaction between dislocations is the main hardening effect. The heat treatment yielded a three-fold increase in the threshold hardness in the Taylor equation, which could have been caused by the annihilation of mobile dislocations and the strengthening effect of oxygen interstitials.

## Figures and Tables

**Table 1:** Abbreviations of the different severe plastic deformation (SPD)-processed and annealed samples used in this study.

Abbreviation	Sample History
1ECAP	1 pass of ECAP
1ECAP–A	1 pass of ECAP + annealing at 430 °C
4ECAP	4 pass of ECAP
4ECAP–A	4 pass of ECAP + annealing at 430 °C
1ECAP–AR	1 pass of ECAP + rolling at ambient temperature
1ECAP–AR–A	1 pass of ECAP + rolling at ambient temperature + annealing at 430 °C
4ECAP–AR	4 pass of ECAP + rolling at ambient temperature
4ECAP–AR–A	4 pass of ECAP + rolling at ambient temperature + annealing at 430 °C
1ECAP–CR	1 pass of ECAP + cryorolling
1ECAP–CR–A	1 pass of ECAP + cryorolling + annealing at 430 °C
4ECAP–CR	4 pass of ECAP + cryorolling
4ECAP–CR–A	4 pass of ECAP + cryorolling + annealing at 430 °C

**Table 2:** The area-weighted mean grain/subgrain size with the misorientation limit of 2° as obtained by EBSD (*d*). The area-weighted mean crystallite size (<*x*>), dislocation density (*ρ*) and arrangement parameter of dislocations (*M*), as obtained by-ray line profile analysis (XLPA).

Sample	*d* [μm]	<*x*> [nm]	*ρ* [10^14^ m^−2^]	*M*
1ECAP	10 ± 2	84 ± 9	17 ± 2	3.3 ± 0.3
1ECAP–A	13 ± 2	88 ± 9	11 ± 1	6.3 ± 0.7
4ECAP	4 ± 1	57 ± 6	25 ± 2	2.2 ± 0.3
4ECAP–A	4 ± 1	82 ± 9	15 ± 2	3.4 ± 0.3
1ECAP–AR	4 ± 1	72 ± 8	16 ± 2	2.4 ± 0.3
1ECAP–AR–A	5 ± 1	112 ± 13	12 ± 1	5.6 ± 0.6
4ECAP–AR	1.8 ± 0.4	65 ± 7	26 ± 3	2.4 ± 0.3
4ECAP–AR–A	2.2 ± 0.5	89 ± 9	15 ± 2	4.3 ± 0.5
1ECAP–CR	2.0 ± 0.2	49 ± 6	40 ± 6	2.2 ± 0.3
1ECAP–CR–A	6 ± 1	61 ± 7	20 ± 2	3.5 ± 0.4
4ECAP–CR	1.3 ± 0.3	55 ± 6	39 ± 5	3.5 ± 0.4
4ECAP–CR–A	1.7 ± 0.4	93 ± 10	25 ± 3	6.4 ± 0.7

## References

[1] Gubicza J., Dobatkin S.V., Khosravi E., Kuznetsov A.A., Lábár J.L. (2011). Microstructural stability of Cu processed by different routes of severe plastic deformation. Mater. Sci. Eng. A.

[2] Islamgaliev R.K., Nesterov K.M., Valiev R.Z. (2015). Structure, strength, and electric conductivity of a Cu-Cr copper-based alloy subjected to severe plastic deformation. Phys. Met. Metallogr..

[3] Shangina D.V., Bochvar N.R., Dobatkin S.V. (2010). Structure and properties of ultrafine-grained Cu-Cr alloys after high pressure torsion. Mater. Sci. Forum.

[4] Rodak K. (2017). Cu-Cr and Cu-Fe alloys processed by new severe plastic deformation: Microstructure and properties. Severe Plastic Deformation Techniques.

[5] Shakhova I., Yanushkevich Z., Fedorova I., Belyakov A., Kaibyshev R. (2014). Grain refinement in a Cu-Cr-Zr alloy during multidirectional forging. Mater. Sci. Eng. A.

[6] Wei W., Wang S.L., Wei K.X., Alexandrov I.V., Du Q.B., Hu J. (2016). Microstructure and tensile properties of Cu-Al alloys processed by ECAP and rolling at cryogenic temperature. J. Alloys Compd..

[7] Gubicza J. (2017). Defect Structure and Properties of Nanomaterials.

[8] Kvackaj T., Kovacova A., Kocisko R., Bidulska J., Litynska–Dobrzynska L., Jenei P., Gubicza J. (2017). Microstructure evolution and mechanical performance of copper processed by equal channel angular rolling. Mater. Charact..

[9] Orłowska M., Bazarnik P., Lewandowska M. (2014). The electrical conductivity of CuCrZr alloy after SPD processing. IOP Conf. Ser. Mater. Sci. Eng..

[10] Kováčová A., Kvačkaj T., Kočiško R., Dragošek L., Lityńska-Dobrzyńska L. (2017). The effect of severe plastic deformation and heat treatment on CuCrZr alloys. Acta Phys. Pol. A.

[11] Lee T.R., Chang C.P., Kao P.W. (2005). The tensile behavior and deformation microstructure of cryo-rolled and annealed pure nickel. Mater. Sci. Eng. A.

[12] Stepanov N.D., Kuznetsov A.V., Salishchev G.A., Raab G.I., Valiev R.Z. (2012). Effect of cold rolling on microstructure and mechanical properties of copper subjected to ECAP with various numbers of passes. Mater. Sci. Eng. A.

[13] Zhu C., Maa A., Jiang J., Li X., Song D., Yang D., Yuan Y., Chen J. (2014). Effect of ECAP combined cold working on mechanical properties and electrical conductivity of conform-produced Cu-Mg alloys. J. Alloys Compd..

[14] Song D., Wang G., Zhou Z., Klu E.E., Gao B., Ma A., Wu Y., Sun J., Jiang J., Ma X. (2020). Developing a high-strength Al–11Si alloy with improved ductility by combining ECAP and cryorolling. Mater. Sci. Eng. A.

[15] Simcák D., Kvackaj T., Kocisko R., Bidulsky R., Kepic J., Puchy V. (2017). Evaluation of hight purity aluminium after asymmetric rolling at ambient and cryogenic temperatures. Acta Metall. Slovaca.

[16] Zhang B., Zhang B. (2018). Physical Fundamentals of Nanomaterials.

[17] Purcek G., Saray O., Nagimov M.I., Nazarov A.A., Safarov I.M., Danilenko V.N., Valiakhmetov O.R., Mulyukov R.R. (2012). Microstructure and mechanical behavior of UFG copper processed by ECAP following different processing regimes. Philos. Mag..

[18] Islamgaliev R.K., Nesterov K.M., Champion Y., Valiev R.Z. (2014). Enhanced strength and electrical conductivity in ultrafine-grained Cu-Cr alloy processed by severe plastic deformation. IOP Conf. Ser. Mater. Sci. Eng..

[19] Kapoor G., Huang Y., Sarma V.S., Langdon T.G., Gubicza J. (2017). Effect of Mo addition on the microstructure and hardness of ultrafine-grained Ni alloys processed by a combination of cryorolling and high-pressure torsion. Mater. Sci. Eng. A.

[20] Straumal B., Kilmametov A., Mazilkin A., Kogtenkova O., Baretzky B., Korneva A., Zieba P. (2019). Diffusive and displacive phase transformations under high pressure torsion. Acta Metall. Slovaca.

[21] Besterci M., Sülleiová K. (2019). Theoretical-experimental possibillities of microstructure quantification of dispersion strengthened materials. Acta Metall. Slovaca.

[22] Sun Y., Peng L., Huang G., Xie H., Mi X., Liu X. (2020). Effects of Mg addition on the microstructure and softening resistance of Cu–Cr alloys. Mater. Sci. Eng. A.

[23] Vinogradov A., Ishida T., Kitagawa K., Kopylov V.I. (2005). Effect of strain path on structure and mechanical behavior of ultra-fine grain Cu-Cr alloy produced by equal-channel angular pressing. Acta Mater..

[24] Purcek G., Yanar H., Saray O., Karaman I., Maier H.J. (2014). Effect of precipitation on mechanical and wear properties of ultrafine-grained Cu-Cr-Zr alloy. Wear.

[25] Dobatkin S.V., Gubicza J., Shangina D.V., Bochvar N.R., Tabachkova N.Y. (2015). High strength and good electrical conductivity in Cu-Cr alloys processed by severe plastic deformation. Mater. Lett..

[26] Urbańczyk-Gucwa A., Rodak K., Płachta A., Sobota J., Rdzawski Z. (2016). Characteristic structure of Cu-0.8Cr alloy using SPD deformation by rolling with cyclic movement of rolls method. Key Eng. Mater..

[27] Xu G.L., Peng L.J., Huang G.J., Xie H.F., Yang Z., Feng X., Yin X.Q., Mi X.J. (2019). Microstructural evolution and properties of a Cu–Cr–Ag alloy during continuous manufacturing process. Rare Met..

[28] Guo X., Xiao Z., Qiu W., Li Z., Zhao Z., Wang X., Jiang Y. (2019). Microstructure and properties of Cu-Cr-Nb alloy with high strength, high electrical conductivity and good softening resistance performance at elevated temperature. Mater. Sci. Eng. A.

[29] Zhang Y., Sun H.L., Volinsky A.A., Tian B.H., Chai Z., Liu P., Liu Y. (2016). Characterization of the hot deformation behavior of Cu-Cr-Zr alloy by processing maps. Acta Metall. Sin..

[30] Fu H., Xu S., Li W., Xie J., Zhao H., Pan Z. (2017). Effect of rolling and aging processes on microstructure and properties of Cu-Cr-Zr alloy. Mater. Sci. Eng. A.

[31] Li R., Guo E., Chen Z., Kang H., Wang W., Zou C., Li T., Wang T. (2019). Optimization of the balance between high strength and high electrical conductivity in CuCrZr alloys through two-step cryorolling and aging. J. Alloys Compd..

[32] Lamperti A., Ossi P.M., Rotshtein V.P. (2006). Surface analytical chemical imaging and morphology of Cu-Cr alloy. Surf. Coatings Technol..

[33] Feng H., Jiang H., Yan D., Rong L. (2019). Thermal stability of ultrafine grained CuCrZr alloy produced by continuous extrusion. Trends J. Sci. Res..

[34] Gubicza J., Hegedús Z., Lábár J.L., Sarma V.S., Kauffmann A., Freudenberger J. (2014). Microstructure evolution during annealing of an SPD- processed supersaturated Cu-3 at.% Ag alloy. IOP Conf. Ser. Mater. Sci. Eng..

[35] Ribárik G., Ungár T., Gubicza J. (2001). MWP-fit: A program for multiple whole-profile fitting of diffraction peak profiles by *ab initio* theoretical functions. J. Appl. Crystallogr..

[36] Gubicza J. (2014). X-ray Line Profile Analysis in Materials Science.

[37] Kamikawa N., Huang X., Tsuji N., Hansen N. (2009). Strengthening mechanisms in nanostructured high-purity aluminium deformed to high strain and annealed. Acta Mater..

[38] Man O., Pantělejev L., Kunz L. (2010). Study of thermal stability of ultrafine-grained copper by means of electron back scattering diffraction. Mater. Trans..

[39] Chengfan G., Tóth L.S., Beausir B., Williams T., Davies C.H.J. (2010). Grain fragmentation in equal channel angular pressed copper. Mater. Sci. Forum.

[40] Stepanov N., Kuznetsov A.V., Salishchev G., Raab G., Valiev R. (2011). Effect of cold rolling on structure and mechanical properties of copper subjected to different numbers of passes of ECAP. Mater. Sci. Forum.

[41] Liu F., Yuan H., Yin J., Wang J.T. (2016). Influence of stacking fault energy and temperature on microstructures and mechanical properties of fcc pure metals processed by equal-channel angular pressing. Mater. Sci. Eng. A.

[42] Gubicza J. (2020). Annealing-induced hardening in ultrafine-grained and nanocrystalline materials. Adv. Eng. Mater..

[43] Valiev R.Z., Sergueeva A.V., Mukherjee A.K. (2003). The effect of annealing on tensile deformation behavior of nanostructured SPD titanium. Scr. Mater..

[44] Gubicza J., Pereira P.H.R., Kapoor G., Huang Y., Vadlamani S.S., Langdon T.G. (2018). Annealing-induced hardening in ultrafine-grained Ni–Mo alloys. Adv. Eng. Mater..

[45] Müllner P., Solenthaler C. (1997). On the effect of deformation twinning on defect densities. Mater. Sci. Eng. A.

[46] Gubicza J., Chinh N.Q., Lábár J.L., Dobatkin S., Hegedus Z., Langdon T.G. (2009). Correlation between microstructure and mechanical properties of severely deformed metals. J. Alloys Compd..

[47] Yu Q., Qi L., Tsuru T., Traylor R., Rugg D., Morris J.W., Asta M., Chrzan D.C., Minor A.M. (2015). Origin of dramatic oxygen solute strengthening effect in titanium. Science.

[48] Guo J., Duarte M.J., Zhang Y., Bachmaier A., Gammer C., Dehm G., Pippan R., Zhang Z. (2019). Oxygen-mediated deformation and grain refinement in Cu-Fe nanocrystalline alloys. Acta Mater..

